# Serum Biomarkers of Brain Injury in Diagnosis of Patients After Seizure in Emergency Department: A Systematic Review

**DOI:** 10.3390/ijms27146432

**Published:** 2026-07-20

**Authors:** Mateusz Antonow, Mariusz Siemiński

**Affiliations:** 1 Department of Emergency Medicine, Medical University of Gdańsk, 80-214 Gdańsk, Poland; antonow@gumed.edu.pl; 2 Emergency Department, University Clinical Centre, 80-214 Gdańsk, Poland

**Keywords:** biomarkers, seizure, epilepsy, syncope, diagnosis

## Abstract

Distinguishing seizures from other causes of transient loss of consciousness in the emergency department (ED) is challenging. This PRISMA-guided systematic review evaluated serum brain injury biomarkers for the acute diagnosis of seizures in adults. We searched PubMed and Web of Science for studies published between 2015 and 2025 and included 14 studies in which blood sampling occurred shortly after the event, reflecting the ED diagnostic window. Given the heterogeneity across studies, the overall certainty of the evidence was low. Neuron-specific enolase (NSE) and ubiquitin carboxyl-terminal hydrolase L1 (UCH-L1) were consistently elevated after epileptic seizures compared to healthy controls. NSE effectively differentiated seizures from syncope, while UCH-L1 and glial fibrillary acidic protein (GFAP) distinguished epileptic from psychogenic non-epileptic seizures (PNESs). Neurofilament light chain (NfL) remained stable after a single seizure but increased markedly in status epilepticus (SE). S100B and BDNF results were inconsistent. Although no single biomarker serves as a standalone test, NSE and UCH-L1 are promising complementary diagnostic tools for identifying epileptic seizures in adults. Furthermore, NfL is a strong candidate marker for SE, reflecting neuroaxonal injury. Larger prospective, standardized studies are needed before routine ED implementation.

## 1. Introduction

Accurate diagnosis of seizure-related presentations in the emergency department (ED) is critical for appropriate treatment and avoiding unnecessary interventions. In practice, differentiating epileptic seizures from non-epileptic events and other causes of transient loss of consciousness (TLOC) remains difficult, particularly in acute settings.

### 1.1. Epidemiology

Syncope is a common clinical problem, affecting up to 30% of the general population at least once in their lifetime and accounting for approximately 0.6–3% of ED visits [1]. Despite a structured evaluation, a definitive cause is identified in only 50–60% of cases after the initial assessment [2]. The differential diagnosis ranges from benign to life-threatening conditions, making safe triage difficult. Accordingly, ED assessment should aim not only to determine the mechanism of syncope but also to rapidly identify patients requiring urgent intervention and those who can be safely discharged versus observed or admitted [3].

Among the many potential TLOC causes that must be considered in this context are epileptic seizures, which are a common reason for ED visits, accounting for approximately 3% of all ED presentations and 1% of hospital admissions [4]. Epilepsy affects an estimated 50 million people worldwide, and the lifetime risk of experiencing at least one seizure is up to 8% [4,5]. Approximately 2.4 million people are newly diagnosed with epilepsy each year, corresponding to one new case every 13 s. Structural causes, including traumatic brain injury and stroke, account for approximately 60% of all cases. Patients with recurrent seizures often have increased healthcare use, excess mortality, and higher rates of physical and psychiatric comorbidities [6].

Non-epileptic seizures are an important clinical problem. Their population incidence is difficult to estimate, but data from epilepsy centers suggest that a substantial proportion of patients referred for refractory epilepsy ultimately have psychogenic non-epileptic seizures (PNESs). In some cohorts, PNESs accounted for up to 26% of such referrals [7]. The diagnosis is often severely delayed; in one study, 20% of patients waited more than 10 years from symptom onset to diagnosis [7].

Status epilepticus is a neurological emergency, with an incidence of up to 61 episodes per 100,000 population per year and an overall mortality rate of approximately 20%, although reported rates range from 1.9% to 40%. The prognosis is closely related to seizure duration, time to recognition, underlying etiology, age, and comorbidity. Early diagnosis and treatment are essential because prolonged seizures increase the risk of irreversible neuronal injury and mortality [8]. Among patients admitted to neurological intensive care units with presumed status epilepticus, 23.1% have been reported to have “pseudostatus epilepticus”, in which the presentation is non-epileptic [7].

### 1.2. Diagnostic Challenges

Even with a structured evaluation, differentiating between syncope, epileptic seizures (including convulsive status epilepticus), and PNES is difficult because all can present with TLOC and motor phenomena. Vasovagal syncope typically includes prodromal autonomic symptoms (e.g., diaphoresis and nausea), brief duration (<15 s), and rapid recovery without persistent neurological deficits. Epileptic seizures more often involve sustained tonic–clonic activity, lateral tongue biting, and a postictal period of confusion or somnolence. Supportive laboratory abnormalities may include elevated lactate or creatine kinase levels. PNES can closely mimic epileptic seizures but lacks ictal EEG discharges, may show variable or asynchronous movements, and typically does not have a true postictal state. Limited eyewitness information and incomplete semiology further increase the risk of misdiagnosis, which has been reported in up to 30% of presumed epilepsy cases [3].

### 1.3. Why Is It Important?

Correct classification of seizure-like events is necessary to guide the diagnostic pathways and acute management. Mislabeling may lead to inappropriate testing, such as neuroimaging when cardiac evaluation is required, or repeated low-yield imaging instead of targeted investigations. It also affects acute treatment decisions, including the avoidance of unnecessary benzodiazepines in PNES and timely escalation in true status epilepticus. Discharge counseling regarding driving, safety, and follow-up differs among epilepsy, syncope, and functional events. Accurate diagnosis can reduce unnecessary resource use, limit exposure to potentially harmful interventions, and mitigate the stigma and social consequences of incorrect epilepsy diagnoses. Therefore, reliable biomarkers that support early differentiation would be clinically valuable [9,10,11].

### 1.4. Why Is It Difficult? What Can Be Done?

The current evaluation of TLOC often relies on neuroimaging and prolonged electroencephalography (EEG) monitoring. Although informative, these tests are time-consuming, costly, and dependent on specialized equipment and staff. Limited access can delay decisions, extend the ED length of stay, and strain resources, particularly in non-tertiary centers without continuous availability of imaging and neurophysiology services [9]. Serum biomarkers can provide a complementary, rapidly available source of objective information, as they can be measured alongside routine laboratory tests within standard turnaround times.

### 1.5. What Is a Biomarker?

A biomarker is a measurable indicator of a biological process or response. The World Health Organization broadly defines biomarkers as functional or physiological responses, cellular biochemical changes, or molecular interactions. In epilepsy, biomarkers can improve diagnostic accuracy, support risk stratification, and help monitor disease activity or treatment response. The lack of reliable biomarkers contributes to diagnostic uncertainty and reliance on subjective reports (e.g., seizure diaries) [9]. Candidate blood biomarkers include neuronal proteins (e.g., S100B and NSE), markers of neuroinflammation (e.g., cytokines such as IL-6 and TNF-α), and oxidative stress mediators. These measures may help distinguish epileptic from non-epileptic events and provide insights into seizure-related injuries and epileptogenesis [9,10].

### 1.6. Biological Rationale: Why Do Biomarkers Change After a Seizure?

Clinically, postictal biomarker changes are usually interpreted as signals of cellular stress and transient blood–brain barrier dysfunction rather than evidence of a single uniform mechanism. Generalized tonic–clonic seizures and status epilepticus can induce metabolic crises, excitotoxicity, and neuroinflammation. These processes may lead to reversible membrane disruption, cytoskeletal injury, and glial activation, which can facilitate the release of intracellular proteins into the interstitial fluid and ultimately into the blood. Sampling time is important because many biomarkers have distinct release and clearance kinetics, which are a major source of between-study variability.

### 1.7. Mechanistic Overview of Biomarkers Discussed in This Review

**GFAP** is an intermediate filament protein that is specific to astrocytes. Elevated serum GFAP levels indicate astroglial injury or activation and are well established in traumatic brain injury (TBI) research [12]. **S100B** is a calcium-binding protein that is predominantly expressed in astrocytes and Schwann cells. Serum S100B is often used as a marker of astroglial injury and blood–brain barrier permeability; however, it is not CNS-specific and can be influenced by extracranial sources [13]. **UCH-L1** is a neuron-enriched deubiquitinating enzyme that is involved in the ubiquitin–proteasome system. Increased serum UCH-L1 levels are considered markers of neuronal cell body injury and may also reflect blood–brain barrier disruption [14]. **NfL** is a structural axonal protein that is released after neuroaxonal injury. In seizure-related presentations, available data suggest that NfL is more strongly associated with prolonged seizures and status epilepticus than with single, isolated events [15]. **NSE** is a cytosolic glycolytic enzyme that is enriched in neurons and neuroendocrine cells. In the context of seizures, higher serum NSE levels are generally interpreted as a marker of neuronal injury or stress, although values can be affected by hemolysis and other preanalytical factors [16]. **BDNF** is a neurotrophin that is involved in synaptic plasticity. Seizure-related changes in circulating BDNF levels may reflect activity-dependent signaling, stress responses, and comorbid factors, which could explain the inconsistent findings [17].

### 1.8. Traumatic Brain Injury (TBI) Biomarker Frameworks and Relevance for Seizure-Related Emergencies

GFAP, UCH-L1, and S100B have the strongest translational track records among the biomarkers discussed here because they have been widely studied in TBI. The recent NIH-NINDS CBI-M framework (Clinical, Biomarker, Imaging, Modifiers) explicitly recommends these blood biomarkers for acute (within 24 h) TBI characterization and uses structured biomarker classification to link the biological source, injury process, and context of use [18]. In TBI care, these biomarkers are typically used to support risk stratification and, in some settings, to guide decisions regarding neuroimaging. Their use has also been informed by structured biomarker classifications that distinguish the biological source (neuronal vs. glial), injury process (structural damage, inflammation, blood–brain barrier disruption), and intended context of use (screening, diagnosis, prognosis, monitoring). Although seizure-related injuries differ from trauma, these conceptual frameworks remain helpful in ED decision-making because they emphasize that a biomarker should not be interpreted in isolation and must be matched to a specific clinical question.

### 1.9. Aim of Study

This review aimed to synthesize evidence on the commonly studied serum biomarkers of brain injury measured shortly after hospital presentation following a seizure, with a focus on their diagnostic value in adult patients. Pediatric studies were excluded from the analysis. Although not all included studies were conducted strictly in ED settings, we prioritized studies that reflected emergency conditions by including only those with blood sampling within a short time window after admission to align with the acute diagnostic needs of emergency care.

## 2. Methods

**Figure 1:**
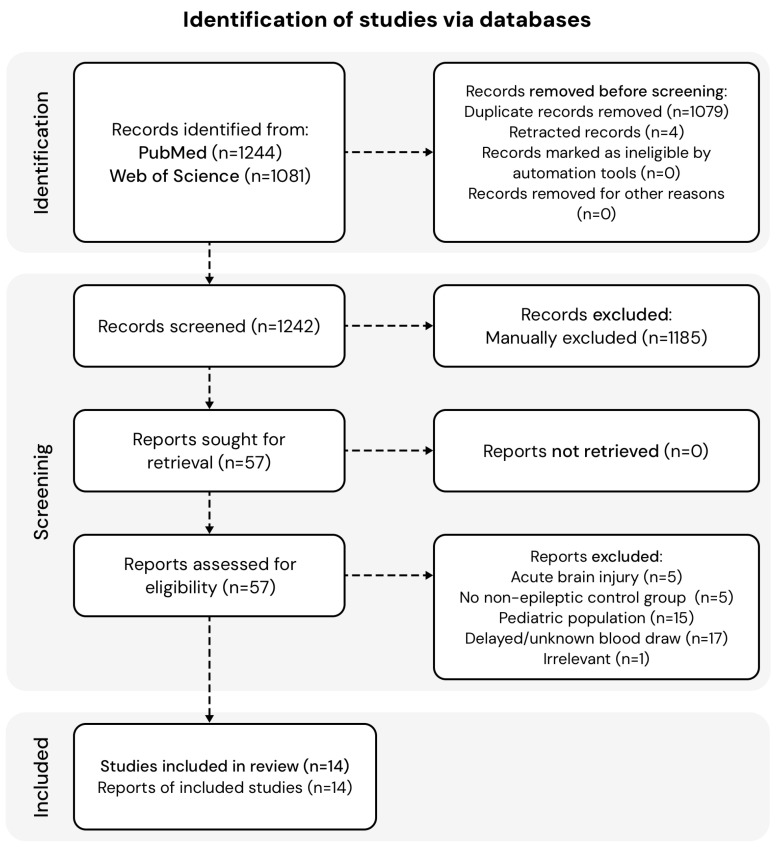
PRISMA 2020 flow diagram illustrating the study selection process.

This systematic review was conducted and reported in accordance with the Preferred Reporting Items for Systematic Reviews and Meta-Analyses (PRISMA) 2020 statement. A completed PRISMA 2020 checklist is provided as Supplementary Material (Supplementary File S1), and the study selection process is summarized in the PRISMA flow diagram (Figure 1). The review question was structured using the PICOTS framework (Patients, Interventions, Comparisons, Outcomes, Timing, Study design) presented in Table 1.

### 2.1. Evidence Acquisition

Databases were searched on the 12 January 2026 to identify eligible studies. The search strategy was as follows: (epilepsy or seizure or (epileptic state) or (non-epileptic seizure)) and (GFAP or S100B or UCH-L1 or NfL or NSE or BDNF). The initial search returned 2325 results. The screening process is illustrated in the PRISMA flowchart (Figure 1). After duplicate removal, the titles and abstracts were screened manually for relevance against the eligibility criteria. All articles marked as relevant by the reviewers were subjected to full-text screening.

### 2.2. Eligibility Criteria

Studies were eligible if they were original, peer-reviewed research articles that measured serum levels of at least one of the predefined brain-injury biomarkers (GFAP, S100B, UCH-L1, NfL, NSE, or BDNF) in adult patients after a seizure, epileptic seizure, psychogenic non-epileptic seizure (PNES), or status epilepticus, and included a comparator group (healthy controls, syncope, or PNES). Only studies in which blood sampling was performed within a short, defined time window after the event were included, in order to reflect the acute diagnostic window of emergency care. Studies were excluded if they (i) enrolled patients with acute brain injury (e.g., stroke, encephalitis, or autoimmune neurological disorders), (ii) lacked a non-epileptic control group, (iii) were conducted in a pediatric population, (iv) did not report the timing of blood sampling or collected samples too long after the event to reflect the immediate postictal state, or (v) were otherwise irrelevant to the research question. No language filters were applied, and the search was limited to articles published between 2015 and 2025.

### 2.3. Selection Process

After the removal of duplicate and retracted records, two reviewers (M.A. and M.S.) independently screened all titles and abstracts against the eligibility criteria. Records considered potentially relevant by either reviewer were retrieved in full text and assessed independently by the same two reviewers for final inclusion. Disagreements at any stage were resolved by discussion until a consensus was reached. No automation tools were used in the selection process.

### 2.4. Data Collection Process and Data Items

Data were extracted independently by two reviewers (M.A. and M.S.) using a standardized, piloted data-extraction form, and any discrepancies were resolved by consensus. No automation tools were used, and study authors were not contacted for additional data. For each included study we extracted the biomarker(s) assessed, first author, year of publication, study centre and country, study population and group sizes, sex distribution, age, the time between the event and blood sampling, and the clinical scenario(s) addressed (seizure vs. syncope; epilepsy vs. non-epilepsy; non-epileptic seizures; epileptic state). The primary outcome was the ability of each serum biomarker to differentiate between the compared groups, assessed through reported between-group differences in biomarker concentrations and, where available, diagnostic accuracy measures (e.g., area under the ROC curve [AUC], sensitivity, and specificity). Where reported, the statistical significance of between-group comparisons was recorded. These variables are summarized in Table 2.

### 2.5. Data Synthesis

Because of the substantial clinical and methodological heterogeneity across the included studies—in particular differences in biomarkers, assays, blood-sampling time windows, comparator groups, and outcome reporting—a quantitative meta-analysis was not appropriate. Results were therefore synthesized narratively and grouped according to four predefined clinical scenarios (seizure vs. syncope; epilepsy vs. non-epilepsy; differentiation of non-epileptic seizures; and diagnosis of the epileptic state). Study characteristics and results were tabulated to allow structured comparison across studies (Table 2). No formal subgroup analysis, meta-regression, or sensitivity analysis was performed given the narrative nature of the synthesis and the limited number of studies per biomarker and scenario.

### 2.6. Risk-of-Bias and Certainty Assessment

The methodological quality and risk of bias of the included studies were appraised independently by two reviewers (M.A. and M.S.) using the QUADAS-2 (Quality Assessment of Diagnostic Accuracy Studies-2) tool [32], which was selected because the primary outcome of this review is the diagnostic accuracy of serum biomarkers. QUADAS-2 evaluates four domains for risk of bias—patient selection, index test, reference standard, and flow and timing—and the first three domains additionally for applicability concerns, each rated as low, high, or unclear. Disagreements between reviewers were resolved by consensus. The certainty of the body of evidence was assessed according to the GRADE approach using the GRADEpro framework (McMaster University and Evidence Prime, 2023. Available from gradepro.org) [33].

### 2.7. Final Selection

Of the 57 reports assessed for eligibility, 43 were excluded for the following reasons: inclusion of patients with acute brain injury (*n* = 5), such as stroke, encephalitis, or autoimmune neurological disorders; lack of a non-epileptic control group (*n* = 5); pediatric study population (*n* = 15); delayed or unknown timing of blood sample collection (*n* = 17); and irrelevance to the research question (*n* = 1). Studies were excluded due to blood sampling issues if they did not report the time of sample collection, collected samples too long after the event to reflect the immediate postictal state, or considered epilepsy as a general condition without examining the direct relationship between biomarker levels and the short time frame after the seizure. This criterion was essential given the focus on identifying biomarkers relevant for differential diagnosis in the emergency department. Ultimately, 14 studies met the inclusion criteria and were included in the final review.

## 3. Results

### 3.1. Clinical Scenarios Included in the Review

Four predefined clinical scenarios were analyzed in this review. In the *epilepsy* vs. *non-epilepsy* scenario, studies compared individuals who experienced a seizure, either a first seizure or in the context of an established epilepsy diagnosis, with a control group of healthy individuals; no further stratification of epilepsy cases by subtype was performed. In the *epilepsy* vs. *syncope* scenario, a single study compared biomarker levels in individuals after an epileptic seizure with those in individuals after syncope; this study also included an additional control group of healthy individuals. In the *non-epileptic seizure* scenario, studies compared biomarker levels in individuals after an epileptic seizure with those in individuals after a psychogenic non-epileptic seizure, and a healthy control group was also included. Finally, in the *epileptic state* scenario, studies assessed biomarkers for diagnosing an ongoing epileptic state in comparison with a healthy control group; in two studies addressing this scenario, biomarker levels were compared with those in patients after an epileptic seizure.

### 3.2. Use of Individual Biomarkers

The use of individual biomarkers in the four predefined clinical scenarios is summarized in Table 2 (Characteristics of the included studies). **S100B** was evaluated in seven studies and was used to differentiate epilepsy from non-epilepsy, identify non-epileptic seizures, and detect the presence of an epileptic state. **NSE** was examined in five studies, focusing on *epilepsy* vs. *non-epilepsy* and *seizure* vs. *loss of consciousness*; notably, it was the only biomarker assessed for distinguishing seizures from syncope. **BDNF** was analyzed in two studies, both addressing *epilepsy* vs. *non-epilepsy*. **NFL**, also reported in two studies, was used to differentiate epilepsy from *non-epilepsy* and in diagnosing an epileptic state. **UCH-L1** appeared in two studies that investigated *epilepsy versus non-epilepsy* and identified non-epileptic seizures. Finally, **GFAP** was reported in a single study that assessed its role in *epilepsy versus non-epilepsy* and in diagnosing non-epileptic seizures.

### 3.3. Epilepsy vs. Non-Epilepsy

All 14 studies addressed the differentiation between epilepsy and healthy controls. Seven of them investigated S100B, five NSE, two UCH-L1, two BDNF, two NFL, and one GFAP.

Yu et al. examined biomarkers NSE, S100B, and Nesfatin-1 using a silver-nanoparticle-based detection assay; as Nesfatin-1 falls outside the biomarker panel defined for this review, only the NSE and S100B findings are discussed here [22]. They measured levels within one hour after a seizure and during the interictal period and compared them with healthy controls. They found significantly higher NSE and S100B levels in the immediate postictal period than in the interictal and control values. Serum S100B and NSE levels were positively correlated with seizure frequency but not with EEG abnormalities. In ROC analysis, the area under the curve (AUC) for S100B and NSE was 0.881 and 0.868, respectively (*p* < 0.05) [22].

Zhang et al. also evaluated NSE and S100B levels, comparing individuals within six hours after a seizure with healthy controls and assessing cognitive function. Serum NSE and S100B levels in patients with epilepsy, with and without cognitive impairment, were significantly higher than those in the control group. Moreover, patients with cognitive impairment had significantly higher serum levels of both biomarkers than those without [21].

Similar conclusions regarding S100B were reached by Cudna et al., who investigated the dynamics of this biomarker. Samples were collected at 1–3, 24, and 72 h post-seizure. Serum S100B levels were significantly higher than those in the controls at all three time points, showing a peak at 24 h, followed by a decline, although the levels remained elevated at 72 h [24].

Tan et al. evaluated NSE in patients sampled 3–72 h post-seizure. Within this window, NSE levels in the epilepsy group were significantly higher than those in the control group, with the difference being the most significant in the 12–48 h subgroup. In ROC analysis, serum NSE showed moderate discrimination (AUC 0.76) with high sensitivity (89.7%) but limited specificity (51.7%) [30].

Consistent with these findings, Masoumi et al. reported significantly higher NSE concentrations in patients after seizures than in healthy controls. By contrast, ROC analysis showed an AUC of 0.973, yielding 93.2% sensitivity and 95.5% specificity, indicating excellent discrimination [31].

Poniatowski et al. evaluated BDNF levels at 1, 3, and 72 h following a seizure, comparing them with healthy controls. The authors observed a significant decrease in BDNF concentration at each time point. In a separate group of patients assessed during the interictal period, serum BDNF concentrations did not differ from those of the control group [27].

Alvin et al. reached different conclusions in their large study on BDNF (*n* = 446). They compared biomarker levels in patients with seizures in the past 24 h and in those who were seizure-free for more than five days, concluding that there was no difference. They reported that BDNF levels were significantly higher in patients with epilepsy than in healthy controls [26].

Two studies by Maiti et al., based on the same cohort, were originally designed to evaluate treatment effectiveness but also assessed NSE and S100B within 48 h of a seizure. A 2017 study found that NSE levels were significantly higher in patients after seizures than in healthy individuals [29]. A 2018 study found that S100B levels were significantly higher after a seizure than in the control group [19]. Moreover, in both studies, biomarkers were effectively used to assess the effectiveness of the treatment.

Two subsequent studies by Giovannini et al. focused on diagnosing status epilepticus but included a post-seizure patient group. In their 2022 study, no differences were found between the NFL levels in patients with epilepsy (enrolled within 24 h of a seizure) and healthy controls [15]. They reached the same conclusion in a 2023 publication based on a different population [23]. The 2023 study also examined S100B and found that its serum levels were significantly higher in the epilepsy group, confirming previous findings [23].

Simani et al. assessed GFAP levels in a study focused on differentiating psychogenic non-epileptic seizures (PNESs). Blood samples collected within six hours of the seizure showed significantly higher serum GFAP levels in patients with epileptic seizures than in healthy controls [28]. Based on the same cohort, Asadollahi and Simani investigated UCH-L1 and S100B and found that the levels of both were significantly higher than those in healthy individuals [20].

Finally, Dapic Ivancic et al. evaluated UCH-L1 and S100B in a study focused on PNESs. They were the only authors to find no significant difference in S100B levels between post-seizure patients and healthy controls. However, they confirmed previous findings by reporting a statistically significant difference in UCH-L1 levels, with significantly higher levels observed in patients after epileptic seizures [25].

### 3.4. Seizure vs. Syncope

We identified a single study that met the predefined criteria and addressed the clinical scenario of seizures versus those of syncope. The study by Masoumi et al. aimed to evaluate the diagnostic value of NSE in differentiating syncope from seizures in patients in the emergency department. The time from seizure onset to blood sampling was not reported; the study only stated that samples were collected upon ED admission and study group inclusion. Patients were differentiated based on their EEG findings. An additional control group consisting of individuals with no history of short-lasting loss of consciousness was included. The NSE level was significantly higher in the seizure group than in the syncope and control groups. No significant difference in NSE levels was observed between the syncope and control groups [31].

### 3.5. Differentiation of Non-Epileptic Seizures

Three studies met the inclusion criteria and investigated the diagnosis of psychogenic non-epileptic seizures. Each of the three studies assessed the use of biomarkers in the differential diagnosis between two study groups: individuals after an epileptic seizure and those with psychogenic non-epileptic seizures (PNES), and a control group consisting of healthy individuals. Each of the three studies had previously been mentioned in the context of epilepsy vs. non-epilepsy clinical scenarios. This section provides further elaboration of the findings related specifically to non-epileptic seizures.

As noted earlier, Simani et al. conducted the only study in this review that investigated the GFAP. Blood samples were obtained within six hours of the event, and PNES was distinguished from epileptic seizures using EEG findings. Patients with epilepsy had significantly higher serum GFAP levels than those in the PNES group and healthy controls. Moreover, there were no statistically significant differences in serum GFAP levels between patients with PNES and healthy controls [28]. In a twin study based on the same cohort, Asadollahi and Simani drew conclusions regarding non-epileptic seizures while evaluating S100B and UCH-L1 levels. Serum S100B concentrations were significantly higher in patients after a seizure than in those with PNES and in healthy controls. Interestingly, patients with PNES had significantly higher serum S100B levels than healthy controls. Serum UCH-L1 concentrations were significantly higher in patients after seizures than in those with PNES and in healthy controls [20].

We also previously mentioned the study by Dapic Ivancic et al., which likewise evaluated the usefulness of UCH-L1 and S100B in the diagnosis of non-epileptic seizures. Epilepsy and PNES were differentiated based on EEG, with blood samples collected between 30 min and 3 h after the event. This study also included a control group of healthy individuals. Patients with epilepsy had significantly higher UCH-L1 levels than those with PNES and healthy controls. No significant differences were observed between the PNES and healthy control groups. Furthermore, there was no significant difference in S100B protein levels among the three groups [25].

### 3.6. Diagnosis of Epileptic State

Finally, we discuss the last clinical scenario, namely the diagnosis of status epilepticus. We identified two publications from the same center. The aim of these studies was to compare biomarker levels in adult patients with status epilepticus, patients with epilepsy, and healthy controls. Expanding on what was discussed in the earlier sections of this review, in their 2022 study, Giovannini et al. investigated NFL levels in the three previously outlined clinical scenarios. Blood samples were collected after diagnosis in the SE group and within 24 h in the epilepsy group. Serum NFL levels were markedly higher in patients with SE than in patients with epilepsy and healthy controls. Moreover, the authors found that NFL levels were higher in cases of SE lasting >24 h than in those lasting 24 h or less. Notably, no correlation was observed between serum NFL levels and the time from the estimated onset of SE to sample collection [15].

One year later, Giovannini et al. published a study involving a different cohort but including the same three groups of patients: those with status epilepticus, those with chronic epilepsy, and healthy controls. Blood samples from the SE group were collected within 72 h of diagnosis. As mentioned previously, the precise timing of blood sampling after an epileptic seizure was not indicated; the authors stated only that the samples were collected after isolated seizures during hospital stay. Patients with status epilepticus (SE) show great variability in serum NFL levels. Nevertheless, the authors confirmed that in the SE group, serum NFL levels were significantly higher than those after epilepsy and healthy controls. However, in contrast to their earlier findings, no differences in serum NFL levels were observed between patients whose samples were collected within 24 h and those collected >24 h after the onset of SE [23]. In this study, S100B levels were also evaluated and were found to be significantly higher in the SE group than in the epilepsy and control groups [23].

### 3.7. Risk of Bias and Certainty of Evidence

Table 3 summarizes the QUADAS-2 risk-of-bias assessment [32] for all 14 included studies; full study-level justifications are provided in Supplementary File S3. Patient selection was rated as a high risk of bias in 13 of the 14 studies (93%), because nearly all studies compared seizure or epilepsy patients with separately recruited healthy volunteers in a case–control design, rather than sampling a consecutive, undifferentiated population presenting with transient loss of consciousness; this pattern is expected to inflate apparent diagnostic accuracy relative to real-world emergency department practice. The index test domain was rated as an unclear or high risk of bias in 13 of 14 studies, mainly because diagnostic thresholds were derived post hoc from receiver operating characteristic analysis on the same sample rather than from pre-specified blinding of biomarker assay interpretation to the clinical or EEG diagnosis. The reference standard (clinical criteria, EEG, or video-EEG) was judged to be appropriate to the target condition in all studies, but blinding of the reference-standard assessment to biomarker results was unclear in 8 of 14 studies. Flow and timing were adequate (low risk) in 7 studies, whereas two studies used markedly heterogeneous sampling windows between the index test and the reference standard. Overall, the included evidence carries a substantial and consistent risk of bias, driven primarily by case–control patient selection and unclear blinding, which—together with the clinical and methodological heterogeneity across studies—supports the overall LOW certainty rating obtained using the GRADEpro approach [33].

## 4. Discussion

This systematic review synthesizes the current evidence on the utility of serum brain injury biomarkers in the acute diagnostic setting of seizure-related conditions. The findings reveal a complex landscape in which some biomarkers show consistent promise for specific clinical scenarios, while others yield conflicting results, underscoring the challenges in establishing a single, universally reliable diagnostic tool.

A key area of consensus is the potential of NSE and UCH-L1 levels. Multiple studies have consistently reported significantly elevated levels of both markers in patients following an epileptic seizure compared to healthy controls. Most notably, NSE was valuable in differentiating seizures from syncope. One study showed significantly elevated levels in patients with seizures, whereas the levels in the syncope group were comparable to those in healthy controls.

Similarly, both UCH-L1 and GFAP showed consistent utility in differentiating epileptic seizures from psychogenic non-epileptic seizures, with studies reporting elevated levels only in the epileptic seizure group, making them valuable candidates for distinguishing between organic and functional events.

However, existing studies have shown conflicting results, particularly regarding S100B and BDNF. While most studies report significantly higher S100B levels after seizures than in controls, Dapic Ivancic et al. [25] found no such difference, challenging this consensus.

This inconsistency was further amplified in the context of PNES, where Asadollahi and Simani [20] found that S100B levels were higher in patients with PNES than in healthy controls, whereas Dapic Ivancic et al. [25] found no differences among any of the three groups (epileptic seizure, PNES, and healthy controls). The findings for BDNF are even more polarized; Poniatowski et al. [27] observed a significant decrease in postictal BDNF levels, whereas Alvim et al. [26] reported that BDNF levels were significantly higher in patients with epilepsy than in controls. These conflicting results suggest that the clinical utility of S100B and BDNF is not yet clearly defined and may be influenced by factors not fully captured in the studies analyzed.

A more complex picture emerges for NFL, which appears to be a marker of seizure severity and duration rather than an isolated event. Two separate studies by the same research group consistently found that NFL levels were not significantly different between patients after a single epileptic seizure and healthy controls. However, in both studies, NFL levels were markedly and significantly elevated in patients with status epilepticus compared to those in both the post-seizure and control groups, positioning it as a powerful biomarker for identifying this neurological emergency. This suggests that a substantial and sustained neuronal injury, which is characteristic of SE, is required to produce a detectable increase in serum NFL levels. A minor inconsistency was noted regarding its correlation with SE duration, which was observed in one study but not in the other.

### Limitations

This review has several limitations. At the level of the evidence, the included studies were mostly small, single-centre, and observational, with marked heterogeneity in the biomarkers studied, assay methods, comparator groups, and—most importantly—the time between the event and blood sampling, which is a major determinant of biomarker concentrations. This heterogeneity precluded a quantitative meta-analysis and limits the strength of the conclusions; accordingly, the overall certainty of the evidence was rated as LOW using the GRADEpro approach. At the level of the review process, only two databases (PubMed and Web of Science) were searched, and grey literature was not included, so relevant studies may have been missed. In addition, a formal assessment of reporting bias (e.g., publication bias) was not possible due to the narrative synthesis and the small number of studies per biomarker, and the review was not prospectively registered. These limitations should be considered when interpreting the findings.

## 5. Conclusions

No serum biomarker currently provides a definitive, standalone diagnosis of epileptic seizures in adults presenting to the ED. Nonetheless, available evidence supports NSE and UCH-L1 as the most promising adjunct biomarkers for postictal evaluation in adults, with NSE also showing potential for differentiating seizures from syncope. Evidence for UCH-L1 and GFAP suggests their potential utility in distinguishing epileptic seizures from PNES, although validation in larger prospective cohorts is required. NfL appears to be a candidate biomarker for identifying seizure-related neuroaxonal injury in status epilepticus rather than in isolated seizures, which may help frame its clinical use as a marker of seizure severity.

Future research should prioritize the validation of these promising markers through large-scale prospective studies with standardized protocols for sample timing and processing to pave the way for their integration into routine clinical practice in the emergency department.

## Figures and Tables

**Table 1:** PICOTS framework used in this systematic review.

Patients	Patients hospitalized following a seizure, epilepsy, psychogenic non-epileptic seizure (PNES), or status epilepticus, from whom blood samples were collected after admission.
Intervention	Measurement of serum biomarkers of brain injury (GFAP, S100B, UCH-L1, NSE, NFL, BDNF) as a diagnostic tool.
Comparison	Healthy individuals.
Outcomes	The ability of biomarkers to differentiate healthy individuals from those with seizure-related conditions.
Time	2015–2025
Study design	Original studies with control groups.

**Table 2:** Characteristics of the included studies.

Biomarker	First Author	Year	Centre	Study Population	M (F)	Age	Time Between Hospital Admission and Blood Administration	Clinical Situation			
								Seizure vs. Syncope	Epilepsy vs. Non-Epilepsy	Non-Epileptic Seizures	Epileptic State
**S100B**	Maiti et al. [19]	2018	Epilepsy clinic of AIIMS, Bhubaneswar, Odisha, India	Total number of 90 patients: carbamazepine group (*n* = 30), oxcarbazepine group (*n* = 30), control group (*n* = 30)	Carbamazepine group: 23 (7), oxcarbazepine group: 21 (9) and control group: unspecified	The carbamazepine group had a mean age of 29.3 ± 8.77 years; the oxcarbazepine group had a mean age of 26.4 ± 8.91 years, and unspecified control group was “aged 18–45”	Within 48 h of a seizure episode		+		
	Asadollahi and Simani [20]	2019	Loghman-Hakim Hospital, Tehran, Iran	Total number of 82 patients: ES group (*n* = 43), PNES group (*n* = 20) and healthy subjects (*n* = 19)	ES group: 24 (19), PNES group: 5 (15) and healthy subjects: 9 (10)	The ES group had a mean age of 30.25 ± 12.13 years, the PNES group had a mean age of 34.00 ± 12.75 years, and the healthy subjects had a mean age of 29.26 ± 6.67 years	Within 6 h of a seizure episode		+	+, ?	
	Zhang et al. [21]	2020	Department of Neurology, Nanchong Central Hospital, Nanchong, China	Total number of 85 patients: epilepsy group (*n* = 65) and control group (*n* = 30)	Epilepsy group: 37 (28) and control group: 17 (13)	The epilepsy group had a mean age of 41.88 ± 8.82 years, and the control group had a mean age of 42.57 ± 9.31 years	Within 6 h of a seizure episode		+		
	Yu et al. [22]	2021	The Sixth Medical Center of The People’s Liberation Army General Hospital, Beijing, China	Total number of 108 patients: epilepsy patients (*n* = 54) and control group (*n* = 54)	Epilepsy patients: 34 (20) and control group: 36 (18)	The epilepsy group (*n* = 54) had a mean age of 28.8 ± 5.4 years, and the control group had a mean age of 27.7 ± 5.8 years	Within 1 h of seizure episode		+		
	Giovannini et al. [23]	2023	Ospedale Civile Baggiovara Hospital, Modena, Italy	Total number of 144 patients: SE group (*n* = 87), PWE group (*n* = 30) and HC group (*n* = 27)	SE group: 33 (54), PWE group: 17 (13) and HC group: 9 (18)	The SE group had a median age of 70 years, the PWE group had a median age of 36 years, and HC group had a median age of 40 years	Within 72 h of the SE diagnosis (median 24 h, IQR 48)		+		+
	Cudna et al. [24]	2023	2nd Department of Neurology in the Institute of Psychiatry and Neurology in Warsaw, Poland	Total number of 100 patients: epilepsy patients (*n* = 50) and control group (*n* = 50)	Epilepsy group: 31 (19) and control group: 26 (24)	The epilepsy group had a mean age of 47 ± 19.7 years, and the control group had a mean age of 43.3 ± 16 years	1–3, 24 and 72 h of seizure episode		+		
	Dapic Ivancic et al. [25]	2025	Department of Neurology, University Hospital Centre Zagreb, Croatia	Total number of 98 patients: ES group (*n* = 32), PNES group (*n* = 36) and healthy controls (*n* = 30)	ES group: 21 (11), PNES group: 5 (31) and healthy controls: 11 (19)	The ES group had a median age of 39 years, PNES group had a median age of 36 years, and healthy controls had a median age of 37 years	30 min to 3 h of seizure episode		−, ?	−, ?	
**BDNF**	Alvim et al. [26]	2021	Epilepsy Outpatient Clinics of the University of Campinas Hospital, Campinas, Brazil	Total number of 612 patients: epilepsy group (*n* = 446) and healthy controls (*n* = 166)	Epilepsy group: 198 (248) and healthy controls: 59 (107)	The epilepsy group had a median age of 44 years, and healthy controls had a median age of 32 years	Patients with seizures <24 h and >5 days were grouped together (no significant BDNF difference observed)		+, ?		
	Poniatowski et al. [27]	2021	2nd Department of Neurology in the Institute of Psychiatry and Neurology in Warsaw, Poland	Total number of 143 patients: aE group (*n* = 50), cE group (*n* = 93) and control group (*n* = 48)	aE group: 31 (19), cE group: 49 (44) and control group: 27 (21)	The aE group had a mean age of 47.0 ± 19.7 years, the cE group had a mean age of 43.3 ± 15.65 years and the control group had a mean age 39.6 ± 13.4 years	1, 3 h and 72 h of seizure episode		+, ?		
**GFAP**	Simani et al. [28]	2018	Epilepsy monitoring unit (EMU) of Loghman-Hakim Hospital, Tehran, Iran	Total number of 82 patients: ES (*n* = 43), PNES (*n* = 20) and control group (*n* = 19)	ES group: 24 (19), PNES group: 5 (15) and healthy subjects: 9 (10)	The ES group had a mean age of 30.25 ± 12.13 years, the PNES group had a mean age of 34.00 ± 12.75 years, and the healthy subjects had a mean age of 29.26 ± 6.67 years	Within 6 h of seizure episode		+	+	
**NSE**	Maiti et al. [29]	2017	Epilepsy Clinic or Psychiatry Outpatient Department of AIIMS, Bhubaneswar, Odisha, India	Total number of 90 patients: carbamazepine group (*n* = 30), oxcarbazepine group (*n* = 30) and healthy controls (*n* = 30)	Carbamazepine group: 23 (7), oxcarbazepine group: 21 (9); no data about control group	The carbamazepine group had a median age of 29.3 ± 8.77 years; oxcarbazepine group had a median age of 26.4 ± 8.91 years; no data about control group	Within 48 h of a seizure episode		+		
	Zhang et al. [21]	2020	Department of Neurology, Nanchong Central Hospital, Nanchong, China	Total number of 85 patients: epilepsy group (*n* = 65) and control group (*n* = 30)	Epilepsy group: 37 (28) and control group: 17 (13)	The epilepsy group had a mean age of 41.88 ± 8.82 years, and the control group had a mean age of 42.57 ± 9.31 years	Within 6 h of a seizure episode		+		
	Tan et al. [30]	2020	Xiangya Hospital, Central South University, Changsha, China	Total number of 87 patients: patient group (*n* = 58) and control group (*n* = 29)	Patient group: 32 (26) and control group: 15 (14)	The patient group had a mean age of 32.25 ± 12.37 years, and the control group had a mean age of 34.52 ± 8.82 years	Within 3–72 h of a seizure episode		+		
	Yu et al. [22]	2021	The Sixth Medical Center of The People’s Liberation Army General Hospital, Beijing, China	Total number of 108 patients: epilepsy patients (*n* = 54) and control group (*n* = 54)	Epilepsy patients: 34 (20) and control group: 36 (18)	The epilepsy group (*n* = 54) had a mean age of 28.8 ± 5.4 years, and the control group had a mean age of 27.7 ± 5.8 years	Within 1 h of seizure episode		+		
	Masoumi et al. [31]	2022	Department of Emergency Medicine, Isfahan University of Medical Sciences, Isfahan, Iran	Total number of 111 patients: syncope (*n* = 44), seizure (*n* = 55) and control group (*n* = 44)	Syncope group: 23 (21), seizure group: 32 (23) and control group: 25 (19)	The syncope group had a mean age of 47.11 ± 14.9 years, seizure group had a mean age of 42.86 ± 11.4 years, and the control group had a mean age of 41.36 ± 12.1 years	Upon ED admission and study group inclusion (time unspecified)	+	+		
**NFL**	Giovannini et al. [15]	2022	Ospedale Civile Baggiovara Hospital, Modena, Italy	Total number of 90 patients: SE group (*n* = 30), epilepsy group (*n* = 30) and healthy controls (*n* = 30)	SE group: 16 (14), epilepsy group: 17 (13), and healthy controls: 10 (20)	The SE group had a mean age of 45 ± 19.9 years, epilepsy group had a mean age of 39 ± 13.6 years, and healthy controls had a mean age of 40 ± 14.7 years	After diagnosis in SE group, <24 h in epilepsy group (median delay 3 h)		−		+
	Giovannini et al. [23]	2023	Ospedale Civile Baggiovara Hospital, Modena, Italy	Total number of 144 patients: SE group (*n* = 87), PWE group (*n* = 30) and HC group (*n* = 27)	SE group: 33 (54), PWE group: 17 (13) and HC group: 9 (18)	The SE group had a median age of 70 years, the PWE group had a median age of 36 years, and HC group had a median age of 40 years	Within 72 h of the SE diagnosis (median 24 h, IQR 48)		−		+
**UCH-L1**	Asadollahi and Simani [20]	2019	Loghman-Hakim Hospital, Tehran, Iran	Total number of 82 patients: ES group (*n* = 43), PNES group (*n* = 20) and healthy subjects (*n* = 19)	ES group: 24 (19), PNES group: 5 (15) and healthy subjects: 9 (10)	The ES group had a mean age of 30.25 ± 12.13 years, the PNES group had a mean age of 34.00 ± 12.75 years, and the healthy subjects had a mean age of 29.26 ± 6.67 years	Within 6 h of a seizure episode		+	+	
	Dapic Ivancic et al. [25]	2025	Department of Neurology, University Hospital Centre Zagreb, Croatia	Total number of 98 patients: ES group (*n* = 32), PNES group (*n* = 36) and healthy controls (*n* = 30)	ES group: 21 (11), PNES group: 5 (31) and healthy controls: 11 (19)	The ES group had a median age of 39 years, PNES group had a median age of 36 years, and healthy controls had a median age of 37 years	30 min to 3 h of seizure episode		+	+	

The plus sign indicates that the results were statistically significant. The minus sign indicates that the results were not statistically different. The question mark indicates that the results were inconsistent when compared with other publications in the same clinical settings. Abbreviations: ES (epilepsy), PNES (psychogenic non-epileptic seizures), PWE (patients with epilepsy), HC (healthy controls), aE (acute epilepsy) [after singular, one to three, GTCS], cE (chronic epilepsy) [enrolled from the outpatient clinic, diagnosed with epilepsy, not immediately after a seizure].

**Table 3:** QUADAS-2 risk-of-bias summary for the 14 included studies.

Study	Patient Selection	Index Test	Reference Standard	Flow and Timing
Cudna et al., 2023 [24]	High	Unclear	Low	Low
Masoumi et al., 2022 [31]	Low	Unclear	Unclear	Unclear
Maiti et al., 2018 [19]	High	Unclear	Low	Low
Maiti et al., 2017 [29]	High	Unclear	Low	Low
Alvim et al., 2021 [26]	High	Low	Low	Unclear
Poniatowski et al., 2021 [27]	High	Unclear	Low	Low
Giovannini et al., 2023 (SE prognosis) [23]	High	Unclear	Low	Low
Zhang et al., 2020 [21]	High	Unclear	Unclear	High
Simani et al., 2018 (GFAP) [28]	High	High	Unclear	Unclear
Giovannini et al., 2022 (NfL) [15]	High	High	Unclear	Unclear
Tan et al., 2020 [30]	High	High	Unclear	High
Yu et al., 2021 [22]	High	High	Unclear	Low
Asadollahi and Simani, 2019 [20]	High	High	Unclear	Unclear
Dapic Ivancic et al., 2025 [25]	High	Unclear	Unclear	Low

Abbreviations: Low (low risk of bias); High (high risk of bias); Unclear (insufficient reporting to judge). Applicability ratings and full per-study justifications are provided in Supplementary File S3.

## Data Availability

All data analyzed in this review were extracted from previously published studies, which are cited in the manuscript. The data-extraction form and the extracted dataset are available from the corresponding author upon reasonable request.

## Supplementary Materials

The following supporting information can be downloaded at: https://www.mdpi.com/article/10.3390/ijms27146432/s1.
