# Supplementary material for: Serum Biomarkers of Brain Injury in Diagnosis of Patients After Seizure in Emergency Department: A Systematic Review
Source: Int J Mol Sci. 2026 Jul 20;27(14):6432. doi: 10.3390/ijms27146432 (PMC13409787; doi:10.3390/ijms27146432)
Supplement: Supplementary file 1 [file ijms-27-06432-s001.zip › Supplementary_File_S2_Review_Protocol .pdf]

Systematic Review Protocol

Serum Biomarkers of Brain Injury in the Diagnosis of Patients after Seizure in the Emergency Department: A Systematic Review

1. Review title

Serum Biomarkers of Brain Injury in the Diagnosis of Patients after Seizure in the Emergency Department: A Systematic Review.

2. Review team / contact

- Mateusz Antonow — Department of Emergency Medicine, Medical University of Gdańsk; Emergency Department, University Clinical Centre, Gdańsk, Poland (corresponding).
- Mariusz Siemiński — Department of Emergency Medicine, Medical University of Gdańsk; Emergency Department, University Clinical Centre, Gdańsk, Poland.

3. Anticipated / actual start and end dates

Searches conducted on 12 January 2026; review restricted to articles published 2015–2025.

4. Funding source

Student Grant programme (SG/01/2024), Medical University of Gdańsk, Poland. The funder had no role in the design, conduct, or reporting of the review.

5. Background and rationale

Differentiating epileptic seizures from other causes of transient loss of consciousness (syncope, psychogenic non-epileptic seizures [PNES], status epilepticus) in the emergency department (ED) is clinically challenging and misdiagnosis is common. Current work-up relies on neuroimaging and EEG, which are costly, time-consuming, and not always rapidly available. Serum brain-injury biomarkers could provide complementary, rapidly available objective information. This review synthesises evidence on serum brain-injury biomarkers measured shortly after seizure presentation, focusing on their diagnostic value in adults.

6. Review question (PICOTS)

- **Population (P):** Adult patients hospitalised following a seizure, epilepsy, PNES, or status epilepticus, with blood sampled after admission.
- **Intervention (I):** Measurement of serum brain-injury biomarkers (GFAP, S100B, UCH-L1, NfL, NSE, BDNF) as a diagnostic tool.

- **Comparison (C):** Healthy individuals (and, where available, syncope or PNES groups).
- **Outcomes (O):** Ability of biomarkers to differentiate seizure-related conditions from comparators (between-group differences; diagnostic accuracy — AUC, sensitivity, specificity where reported).
- **Timing (T):** 2015–2025; blood sampling within a short, defined window after the event.
- **Study design (S):** Original studies with a control group.

7. Condition / domain

Seizure-related presentations in the emergency / acute-care setting; serum neuronal and glial injury biomarkers.

8. Type of review

Systematic review (narrative synthesis; no meta-analysis planned due to anticipated heterogeneity).

9. Eligibility criteria

Inclusion: original peer-reviewed studies; adult population; measurement of ≥1 predefined biomarker (GFAP, S100B, UCH-L1, NfL, NSE, BDNF); presence of a non-epileptic comparator group; blood sampling within a short, defined time window after the event; published 2015–2025.

Exclusion: acute brain injury (stroke, encephalitis, autoimmune neurological disorders); no non-epileptic control group; pediatric population; missing/delayed blood-sampling timing not reflecting the immediate postictal state; irrelevant to the research question.

10. Information sources

PubMed and Web of Science. No registers, trial databases, reference-list searching, or grey-literature sources were used. Last searched: 12 January 2026.

11. Search strategy (full)

```
(epilepsy OR seizure OR (epileptic state) OR (non-epileptic seizure)) AND (GFAP OR S100B OR UCH-L1 OR NfL OR NSE OR BDNF)
```

No filters applied other than publication years 2015–2025. Initial yield: 2325 records.

12. Study selection (screening) process

After removal of duplicate and retracted records, two reviewers (M.A., M.S.) independently screened titles/abstracts against eligibility criteria. Potentially relevant records were retrieved in full text and independently assessed by both reviewers. Disagreements resolved by consensus. No automation tools used.

13. Data extraction (selection and coding)

Data extracted independently by two reviewers (M.A., M.S.) using a standardised piloted form; discrepancies resolved by consensus. Extracted items: biomarker(s); first author; year; centre/country; population and group sizes; sex; age; time from event to blood sampling; clinical scenario; between-group differences and diagnostic accuracy (AUC/sensitivity/specificity) where reported; statistical significance.

14. Outcomes

Primary: ability of each serum biomarker to differentiate compared groups (between-group biomarker differences; diagnostic accuracy where available). Analysed within four predefined clinical scenarios: (i) seizure vs. syncope; (ii) epilepsy vs. non-epilepsy; (iii) differentiation of non-epileptic seizures; (iv) diagnosis of the epileptic state.

15. Risk of bias (quality) assessment

Methodological quality / risk of bias appraised by two reviewers independently, with disagreements resolved by consensus.

16. Strategy for data synthesis

Narrative synthesis grouped by the four predefined clinical scenarios, with tabulation of study characteristics and results. Quantitative meta-analysis not planned owing to clinical and methodological heterogeneity (biomarkers, assays, sampling windows, comparators, outcome reporting). No subgroup analysis, meta-regression, or sensitivity analysis planned.

17. Certainty of evidence

Certainty of the body of evidence assessed using the GRADE approach (GRADEpro).

18. Stage of the review at submission

> **Important for OSF (not PROSPERO):** because data extraction and analysis are complete, this can only be registered retrospectively (e.g., on OSF Registries, which accepts and flags retrospective registrations). PROSPERO does not accept reviews that have already completed data extraction.

Review stage	Started	Completed
Preliminary searches	Yes	Yes
Piloting of the study selection process	Yes	Yes
Formal screening against eligibility criteria	Yes	Yes
Data extraction	Yes	Yes
Risk of bias (quality) assessment	Yes	Yes
Data analysis	Yes	Yes

19. Conflicts of interest

The authors declare no conflict of interest.
