# Supplementary material for: Serum Biomarkers of Brain Injury in Diagnosis of Patients After Seizure in Emergency Department: A Systematic Review
Source: Int J Mol Sci. 2026 Jul 20;27(14):6432. doi: 10.3390/ijms27146432 (PMC13409787; doi:10.3390/ijms27146432)
Supplement: Supplementary file 1 [file ijms-27-06432-s001.zip › Supplementary_File_S3_QUADAS2 .pdf]

Supplementary File S3. QUADAS-2 Risk-of-Bias and Applicability Assessment of Included Studies

Systematic review: Serum Biomarkers of Brain Injury in Diagnosis of Patients after Seizure in Emergency Department: A Systematic Review

Method: Risk of bias and applicability concerns were assessed independently by two reviewers (M.A. and M.S.) using the QUADAS-2 (Quality Assessment of Diagnostic Accuracy Studies-2) tool [19], with disagreements resolved by consensus. Four domains were rated for risk of bias — Patient Selection, Index Test, Reference Standard, and Flow and Timing — and the first three domains were additionally rated for applicability concerns. Each domain was rated as Low, High, or Unclear. Reference numbers correspond to the reference list of the main manuscript.

1. Cudna et al., 2023 [23] — Changes in serum blood-brain barrier markers after bilateral tonic-clonic seizures (Seizure: European Journal of Epilepsy)

Domain	Risk of Bias	Applicability	Justification
1. Patient Selection	High	High	Case-control design: a research group of consecutively admitted patients was compared against a control group of ambulatory neurology patients and healthy volunteers matched for age/sex, not consecutively enrolled ED patients undergoing differential diagnosis.
2. Index Test	Unclear	High	ELISA thresholds were not pre-specified for diagnostic classification (group-level mean differences reported, not a diagnostic cut-off); no statement that laboratory personnel were blinded to case/control status.
3. Reference Standard	Low	Low	Diagnosis based on epilepsy diagnosis or first bilateral tonic-clonic seizure confirmed by a reliable witness — a standard clinical approach, independent of biomarker results.
4. Flow and Timing	Low	—	Blood drawn at pre-specified intervals (1–3 h, 24 h, 72 h); clear flow diagram accounting for all patients.

2. Masoumi et al., 2022 [25] — Differential Diagnosis of Seizure and Syncope by the Means of Biochemical Markers in Emergency Department Patients (International Journal of Preventive Medicine)

Domain	Risk of Bias	Applicability	Justification
1. Patient Selection	Low	Low	Patients recruited from a defined ED population (short-lasting loss of consciousness) classified by EEG into seizure vs. syncope — matches the review's target ED population.
2. Index Test	Unclear	Low	Biomarkers measured at admission, but no statement of blinding to EEG/clinical diagnosis; diagnostic cut-offs (e.g., NSE 25.12) derived post hoc from ROC on the same dataset.

Domain	Risk of Bias	Applicability	Justification
3. Reference Standard	Unclear	Low	EEG performed several times over a long period; appropriate but imperfect reference standard; no statement of blinding to biomarker results.
4. Flow and Timing	Unclear	—	Interval between blood draw (at admission) and final EEG-based classification not clearly reported; 8 of 111 screened patients did not undergo EEG, without explanation.

3. Maiti et al., 2018 [29] — Effect of anti-seizure drugs on serum S100B in patients with focal seizure: a randomized controlled trial (Journal of Neurology)

Domain	Risk of Bias	Applicability	Justification
1. Patient Selection	High	High	60 focal-seizure patients from an epilepsy clinic (not consecutive ED) vs. 30 healthy volunteers — case-control design.
2. Index Test	Unclear	Unclear	S100B cut-off (62 pg/mL) derived via ROC/logistic regression on the same sample; no statement of blinding of assay to clinical/EEG diagnosis.
3. Reference Standard	Low	Unclear	Clinical diagnosis of focal seizure supported by EEG; outpatient epilepsy-clinic recruitment limits applicability to an acute ED PICOTS.
4. Flow and Timing	Low	—	Patients enrolled within 48 h of a seizure episode; CONSORT diagram accounts for all screened/randomized/analyzed patients.

4. Maiti et al., 2017 [28] — Effect of carbamazepine and oxcarbazepine on serum neuron-specific enolase in focal seizures: A randomized controlled trial (Epilepsy Research)

Domain	Risk of Bias	Applicability	Justification
1. Patient Selection	High	High	Same trial infrastructure as study 3 (NCT02705768): 60 focal-seizure outpatients vs. 30 healthy volunteers — case-control design.
2. Index Test	Unclear	Unclear	NSE measured by ELISA; no blinding statement; no pre-specified diagnostic threshold (group means only).
3. Reference Standard	Low	Unclear	Clinical diagnosis of focal seizure within 48 h of presentation; outpatient recruitment reduces applicability to an ED PICOTS.
4. Flow and Timing	Low	—	Enrollment within 48 h of seizure; CONSORT diagram accounts for all screened/randomized/lost-to-follow-up patients.

5. Alvim et al., 2021 [27] — Inflammatory and neurotrophic factor plasma levels are related to epilepsy independently of etiology (Epilepsia)

Domain	Risk of Bias	Applicability	Justification
1. Patient Selection	High	High	Consecutive chronic epilepsy outpatients vs. separately recruited healthy controls (n=166) — case-control design; chronic outpatient population, not acute ED presentation.
2. Index Test	Low	High	Assay analysis explicitly blinded (performed at an independent laboratory); however, sampling context (chronic, non-acute) limits applicability.
3. Reference Standard	Low	Unclear	ILAE criteria plus MRI/EEG — robust and appropriate, but classification reflects chronic disease status rather than acute presentation.
4. Flow and Timing	Unclear	—	No fixed interval specified between (potentially years-prior) diagnosis and index-test blood draw for the main comparison.

6. Poniatowski et al., 2021 [26] — Kinetics of serum brain-derived neurotrophic factor (BDNF) concentration levels in epileptic patients after generalized tonic-clonic seizures (Epilepsy Research)

Domain	Risk of Bias	Applicability	Justification
1. Patient Selection	High	Low	Acute epilepsy group, chronic epilepsy group, and a control group of healthy volunteers/non-epilepsy patients — case-control design; acute-seizure subgroup otherwise maps well to the review's PICOTS.
2. Index Test	Unclear	Low	BDNF measured by ELISA (standardized kit); no statement of blinding to case/control status; no pre-specified diagnostic threshold.
3. Reference Standard	Low	Low	Epilepsy diagnosis or first GTCS confirmed by a reliable witness — standard, appropriate, applied independently of biomarker results.
4. Flow and Timing	Low	—	Blood collected at pre-specified times (1–3 h and 72 h after seizure); flow chart accounts for all groups.

7. Giovannini et al., 2023 [30] — Neuro-glial degeneration in Status Epilepticus: serum Neurofilament light chains and S100B as prognostic biomarkers (Epilepsy & Behavior)

Domain	Risk of Bias	Applicability	Justification
1. Patient Selection	High	Low	Prospective SE registry cohort (consecutive) but explicitly case-control matched against separately recruited healthy controls and epilepsy-after-isolated-seizure patients.
2. Index Test	Unclear	Low	NfL/S100B measured with validated automated immunoassays; retrospective assessment of stored samples; no statement of blinding to clinical status.
3. Reference Standard	Low	Low	ILAE-based SE definition, reviewed by two authors for pre-ILAE cases; rigorous and appropriate.
4. Flow and Timing	Low	—	All samples acquired within 72 h of SE diagnosis (median 24 h); no difference in biomarker levels by early vs. late sampling.

8. Zhang et al., 2020 [22] — Relationship of serum ATPase activity and levels of NSE, S100B and Bcl-2 with cognitive function after epileptic seizure (Annals of Palliative Medicine)

Domain	Risk of Bias	Applicability	Justification
1. Patient Selection	High	High	65 epilepsy patients vs. 30 healthy volunteers, no statement of consecutive/random sampling; restrictive exclusions produce a cleaner population than a typical ED cohort.
2. Index Test	Unclear	Unclear	NSE, S100B, Bcl-2 by ELISA; correlations with cognitive scores reported rather than a pre-specified diagnostic threshold; no blinding statement.
3. Reference Standard	Unclear	Low	Diagnosis via clinical exam + CT/MRI + EEG per 1989 ILAE criteria — standard, but no blinding of EEG/clinical readers to biomarker results.
4. Flow and Timing	High	—	Epilepsy group sampled within 6 h of the attack; healthy controls sampled in an unrelated early-morning resting state — a non-comparable sampling context between groups.

9. Simani, Elmi & Asadollahi, 2018 [31] — Serum GFAP level: a novel adjunctive diagnostic test to differentiate epileptic seizures from psychogenic attacks (Seizure: European Journal of Epilepsy)

Domain	Risk of Bias	Applicability	Justification
1. Patient Selection	High	Low	63 consecutive EMU patients (43 ES + 20 PNES) vs. 19 separately recruited healthy volunteers — case-control overall, though the ES/PNES/EMU population matches PICOTS well.
2. Index Test	High	Low	GFAP cut-off (2.71 ng/mL) derived from the study's own ROC curve (AUC only 0.68); no blinding statement.
3. Reference Standard	Unclear	Low	Video-EEG interpreted by an epileptologist — appropriate reference standard; blinding to GFAP results not stated.
4. Flow and Timing	Unclear	—	Patients sampled within 6 h of seizure; authors themselves flag lack of standardization of sample-collection timing as a limitation.

10. Giovannini et al., 2022 [15] — Serum neurofilament light as biomarker of seizure-related neuronal injury in status epilepticus (Epilepsia)

Domain	Risk of Bias	Applicability	Justification
1. Patient Selection	High	Unclear	Retrospective, biobank-derived, three-arm case-control (30 SE, 30 drug-resistant epilepsy, 30 healthy controls); SE cases with structural/inflammatory/neurodegenerative disease excluded, reducing generalizability.
2. Index Test	High	Low	NfL cut-off (28.8 pg/mL) determined via ROC/Youden index on the same sample; no blinding statement.

Domain	Risk of Bias	Applicability	Justification
3. Reference Standard	Unclear	Low	ILAE 2015 operational definition, reviewed by two authors — appropriate; blinding of reviewers to NfL levels not stated.
4. Flow and Timing	Unclear	—	Sampling within 48 h of SE diagnosis but split roughly 47%/53% before/after 24 h; no correlation found between delay and NfL level.

11. Tan et al., 2020 [24] — Serum Visinin-Like Protein 1 Is a Better Biomarker Than Neuron-Specific Enolase for Seizure-Induced Neuronal Injury (Frontiers in Neurology)

Domain	Risk of Bias	Applicability	Justification
1. Patient Selection	High	Low	Prospective case-control: 58 epilepsy patients vs. 29 matched healthy controls; enrollment method (consecutive/random) not stated.
2. Index Test	High	Low	VILIP-1/NSE/CAV-1 thresholds derived from ROC on the same dataset (AUC 0.93 and 0.76); no blinding statement.
3. Reference Standard	Unclear	Low	Diagnosis per latest ILAE criteria — appropriate; blinding of diagnosing clinician to biomarker results not stated.
4. Flow and Timing	High	—	Wide, heterogeneous sampling window (3–72 h post-seizure, split into 4 subgroups) — explicitly acknowledged by the authors as a limitation.

12. Yu, Liu & Sun, 2021 [21] — Clinical value of EEG monitoring and silver nanoparticles to detect serum Nesfatin-1, S100β, and NSE in evaluating severity and prognosis of epilepsy (Materials Express)

Domain	Risk of Bias	Applicability	Justification
1. Patient Selection	High	Unclear	54 epilepsy patients (further split into seizure/non-seizure states) vs. 54 healthy controls; enrollment not described as consecutive/random; several exclusions narrow the population.
2. Index Test	High	High	Nesfatin-1, S100β, and NSE measured via a novel silver-nanoparticle-assisted ELISA — an experimental, non-routine assay platform (applicability concern); ROC-derived AUCs (0.850/0.881/0.868) used post-hoc thresholds; no blinding statement.
3. Reference Standard	Unclear	Low	ILAE clinical criteria plus neuroimaging/EEG — appropriate; EEG abnormality was not significantly correlated with biomarker levels, an internal inconsistency worth noting; blinding not stated.
4. Flow and Timing	Low	—	Seizure-period sampling within 1 h of onset; non-seizure sampling monthly — clear, well-defined intervals.

13. Asadollahi & Simani, 2019 [32] — Diagnostic value of serum UCH-L1 and S100-B levels in differentiating epileptic seizures from psychogenic attacks (Brain Research)

Note: Same underlying cohort as study 9 [31] (63 EMU patients + 19 healthy controls, Tehran), differing only in the biomarkers analyzed.

Domain	Risk of Bias	Applicability	Justification
1. Patient Selection	High	Low	63 consecutive EMU patients vs. 19 separately recruited healthy controls — case-control overall; ES/PNES/EMU population matches PICOTS well.
2. Index Test	High	Low	UCH-L1 cut-off (5.73 ng/mL) derived via ROC on the study's own data (AUC only 0.71 vs. healthy, 0.68 ES vs. PNES); no blinding statement.
3. Reference Standard	Unclear	Low	Video-EEG interpreted by an epileptologist — appropriate; blinding to biomarker results not stated.
4. Flow and Timing	Unclear	—	Patients sampled within 6 h of a habitual seizure; same lack-of-standardization concerns as the companion GFAP study apply.

14. Dapic Ivancic et al., 2025 [33] — The role of UCH-L1 and protein S100B in differentiating epileptic and psychogenic non-epileptic seizures — Pilot study (Epilepsia Open)

Domain	Risk of Bias	Applicability	Justification
1. Patient Selection	High	Unclear	Prospective three-arm case-control (32 ES, 36 PNES, 30 healthy controls); ES/PNES groups restricted to normal brain MRI only, explicitly excluding structural epilepsy — the authors themselves note this limits generalizability.
2. Index Test	Unclear	Low	S100B by ECLIA, UCH-L1 by ELISA; results emphasize group-comparison p-values rather than a pre-specified diagnostic threshold; no blinding statement.
3. Reference Standard	Unclear	Low	ES diagnosed per ILAE criteria; PNES confirmed by video-EEG evaluated by an epileptologist — appropriate; blinding not stated.
4. Flow and Timing	Low	—	Blood sampling standardized to 30 min–3 h post-seizure based on biomarker half-lives; median 35 min (ES) / 30 min (PNES), no significant between-group difference — the best-controlled timing among all 14 studies.

Cross-Study Summary

Domain	High risk	Unclear	Low risk
1. Patient Selection	13/14 (93%)	0	1/14 (Masoumi et al. [25])
2. Index Test	5/14	8/14	1/14 (Alvim et al. [27])
3. Reference Standard	0	8/14	6/14
4. Flow and Timing	2/14	5/14	7/14

Recurring patterns:

1. **Case-control patient selection (dominant concern).** Thirteen of fourteen studies compared seizure/epilepsy patients against separately recruited healthy volunteers rather than sampling a consecutive, undifferentiated population presenting with transient loss of consciousness or a seizure-like event. This design is expected to inflate apparent diagnostic accuracy relative to real-world emergency department differential diagnosis.
2. **Unreported blinding and post-hoc diagnostic thresholds.** With the sole exception of Alvim et al. [27], no study reported that biomarker assay interpretation was performed blind to the clinical/EEG diagnosis. Most studies reporting diagnostic accuracy metrics (AUC, sensitivity, specificity) derived their cut-off values via ROC/Youden-index analysis on the same sample rather than pre-specifying them.
3. **Variable sampling-to-diagnosis timing.** Intervals ranged from a tightly controlled 30 min–3 h window (Dapic Ivancic et al. [33]) to a markedly heterogeneous 3–72 h window (Tan et al. [24]), with two studies (Zhang et al. [22]; Tan et al. [24]) sampling comparator groups under non-comparable conditions.

These findings are consistent with, and support, the LOW certainty-of-evidence rating obtained using the GRADEpro approach for this systematic review [20].

Prepared by two reviewers (M.A. and M.S.) based on full-text review of all 14 included studies; disagreements resolved by consensus.
